# Core components of an effective pain management education programme for surgical nurses: A Delphi study*

**DOI:** 10.1080/17482631.2022.2110672

**Published:** 2022-08-17

**Authors:** Manaporn Chatchumni, Henrik Eriksson, Monir Mazaheri

**Affiliations:** a School of Nursing, Rangsit University, Pathumthani, Thailand; b Department of Health Sciences, The Swedish Red Cross University, Stockholm, Sweden; c Department of Health Sciences, University West, Trollhättan, Sweden; d Department of Nursing Science, Sophiahemmet University, Stockholm, Sweden; e Department of Neurobiology, Care Sciences and Society, Karolinska Institute, Stockholm, Sweden

**Keywords:** Delphi method, nurse education, pain management education programs, Post-operative pain

## Abstract

The critical reason for this study is the inadequate training received by surgical nurses in acute and chronic pain management nursing services for patients.

**Purpose:**

This study aimed to describe the core components of an effective pain management education programme (PMEP) for surgical nurses in Thailand.

**Methods:**

A three-round Delphi method was used. A panel of 40 experts advised regarding the essential components of an effective PMEP for surgical nurses.

**Results:**

The core components of a PMEP were derived from experts’ panel consensus: (i) multidisciplinary collaboration, (ii) acquisition of innovative knowledge and training by healthcare teams, and (iii) consideration of individual differences when delivering pain management services. To enhance their pain management practices, nurses should adopt multimodal pain approaches that involve family roles and engage in active patient listening.

**Conclusions:**

The PMEP designed in this study, which adheres to international nursing training standards, promotes the competency of professional nurses.

## Introduction

Although a considerable amount of research has been conducted in both low- and high-income countries on pain management education programmes (PMEPs) for nurses, the knowledge level of surgical nurses remains inadequate (Hyland et al., 2021; Jaleta et al., 2021; Keen et al., 2017; Kwon et al., 2020; Al Samaraee et al., 2010). A study conducted in north-western Ethiopia demonstrated that 43.46% of the nurses who participated in the study lacked adequate knowledge regarding pain management (Jaleta et al., 2021). Post-operative pain is poorly managed in clinical practice compared with other clinical procedures (Hyland et al., 2021; Jaleta et al., 2021; Keen et al., 2017; Kwon et al., 2020). Several factors prevent nurses from obtaining an adequate level of pain management knowledge, and this inadequacy is a major hurdle in effective post-operative pain management. Specifically, there is a lack of knowledge and appropriate attitudes regarding effective pain assessment and management by physicians and nurses. In addition, knowledge regarding the effectiveness of multidisciplinary team communication is also inadequate (Coll & Jones, 2020; Jaleta et al., 2021; Ramamoorthy, 2021; Al Samaraee et al., 2010; Tano et al., 2021). A PMEP considers the elements required for post-operative pain management and incorporates updated evidence-based policies and guidelines into clinical practice (Coll & Jones, 2020; Jaleta et al., 2021; Ramamoorthy, 2021; Al Samaraee et al., 2010; Tano et al., 2021). The purpose of such a program is to improve nurses’ pain management competence, that is, their competence in reducing pain levels and morbidity and improving recovery rates (Coll & Jones, 2020; Jaleta et al., 2021; Ramamoorthy, 2021; Al Samaraee et al., 2010; Tano et al., 2021).

However, the number of evidence-based PMEPs developed with the help of expert panels is limited (Basinska et al., 2021; Hu et al., 2017; Sasahara et al., 2009; Sharpe et al., 2020). Some perspectives have been developed through the Delphi method. For example, Basinska et al. (2021) found that educating nurses can improve their competencies in expanded roles and support care teams. Another example is an education programme for anaesthesia nurses developed by Hu et al. (2017). The Delphi method was used to develop an education programme based on international educational standards considering the national context as well as determining the scope of practice and competencies for anaesthesia nurses (Hu et al., 2017). The Delphi method involves iteration steps to collect and condense anonymous expert judgements (Basinska et al., 2021). Data collection and analysis techniques utilizes responses from participants in several rounds of questionnaires where the responses are summed up and shared with the group of participants after each round. The Delphi method is particularly appropriate to improve our understanding of the core components of an effective pain management training programme for surgical nurses. In our study, the Delphi method was chosen because it is characterized by thoroughness and includes a consensus-based approach regarding a given area of uncertainty or a lack of empirical evidence (Karamitri et al., 2013; Worrell et al., 2013).

The review of literature in the Thai context justifies the need to reach a consensus regarding the components that ensure the effectiveness of PMEPs to help surgical nurses in their practice. Previous studies have highlighted the complex communication structure used in surgical wards in Thailand to detect and manage post-operative pain (Chatchumni et al., 2016a). The complexity comprises the involvement of intermediaries (parents and caregivers) in conveying communication between patients and nurses/physicians, instead of direct communication between the care recipients and care providers. Another aspect of complexity is that nurses are required to extensively document post-operative pain and monitor at short intervals which limits the time and attention they can put into providing direct care (Chatchumni et al., 2016a). Another study on Thai surgical wards showed that the primary focus on excessive monitoring and registering patients’ pain through different instruments detract the nurses from the significance of patients’ self-reported pain (Chatchumni et al., 2016b). Thai surgical nurses have been reported to use their own experiences in managing patients’ pain, and some nurses perceived this pain as something that patients needed to endure without treatment (Chatchumni et al., 2015). Overall, surgical nurses were observed to have a passive approach to pain management (Chatchumni et al., 2016a,b). Therefore, there is a need to shift to an evidence-based paradigm to help surgical nurses in Thailand manage post-operative pain (Chatchumni et al., 2016b, 2018, 2019, 2015).

A literature review (Chatchumni, Eriksson, & Mazaheri, 2020) showed that PMEP utilizes diverse methods, including computer-based simulation, web-based facilitation, and video materials for educating nursing students. These methods improve nurses’ competence by enhancing their critical thinking, leadership, patient management, and health-promotion skills. The literature review suggested that PMEPs can help promote new opportunities for collaboration in multidisciplinary team projects (Chatchumni, Eriksson, & Mazaheri, 2020).

### Study purpose

This study adopted the Delphi method to describe the core components of an effective PMEP for surgical nurses in Thailand.

## Material and methods

**Figure 1.**
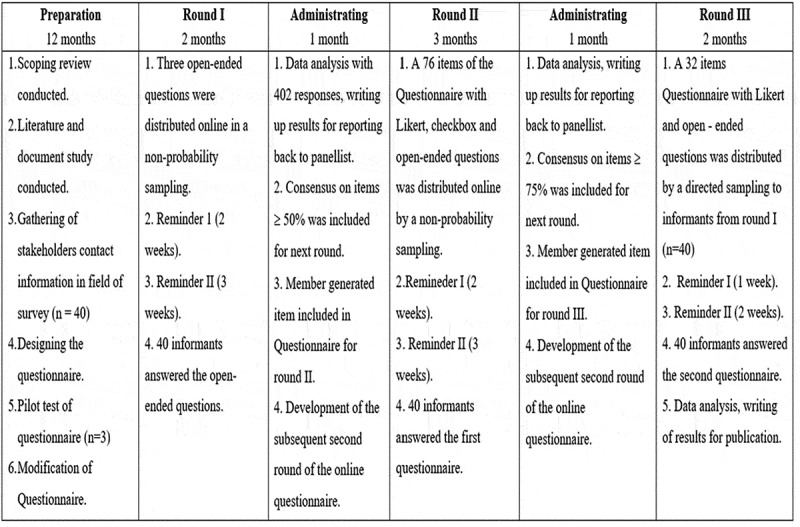
The Delphi processes.

A three-round Delphi method was used in this study. The Delphi method was considered suitable for this study, as there is insufficient information regarding the impact of ineffective pain management in nursing practices and a lack of empirical evidence regarding the same. A consensus needs to be reached among nursing experts and educators regarding the components of a PMEP. The present study aims to achieve a consensus of opinions and central ideas based on Keeney et al. (2011), and consensus agreements among the panel of experts was generated through several series of questionnaires. Expert evaluations are more precise and credible in specific areas than general views. Thus, the Delphi technique is also well-suited when there is a lack of available research evidence in a particular area. This method includes three stages, as illustrated in Figure 1.

### Data collection methodology

We conducted expert panel selection by inviting 40 experts (Table I) via purposeful sampling. These included university lecturers, researchers, and clinical nurses with rich knowledge in the domain of pain management. All experts were nurses, lecturers, and researchers associated with pain management practices; the different levels of skilled practitioners included novice, advanced beginner, competent, proficient, or expert nurses (Benner et al., 2009). All the participants agreed to complete a questionnaire that included three open-ended questions. The authors asked the participants to repeat their answers over a period during the first round of the process. Fishman et al. (2013) categorized consensus competence into the following four areas: the multidimensional nature of pain, pain evaluation and measurement, pain management, and the context of pain management. The open-ended questions were inspired by the Delphi study by Kalamatri et al. (2013). The instructions were as follows:*1) Name the things that make a PMEP feasible and efficient for nurses. 2) In your opinion, what are the main components of a PMEP for nurses? 3) Name at least three elements that could help nurses improve their competence in pain management*.

**Table I.** Demographic and societal characteristics of the experts’ panel.

	n	%	Min	Max	Mean	SD
Age			30	68	44.75	8.53
Gender						
Male	6	15.00				
Female	34	85.00				
Total	40	100.00				
Occupation						
University lecturers/researchers	27	67.50				
Clinical nurses						
Semi-ICU	3	7.50				
Surgical ward	3	7.50				
ICU	2	5.00				
*Nurse Anaesthetists*	2	5.00				
OPD-Medical	1	2.50				
Medical ward	2	5.00				
Total	40	100.00				
Education						
Master of Science in Nursing (MScN)	26	65.00				
Doctor of Philosophy (candidate)	6	15.00				
Doctor of Philosophy (Ph.D.)	8	20.00				
Total	40	100.00				
Experience in nursing care (years)			6	45	20.37	7.63
Special education and training in pain management						
Yes	6	15.00				
No	34	85.00				
Total	40	100.00				

### Characteristics of participants

The participants were selected based on at least 6 years of nursing experience in working with patients with pain. The 40 participants were working full-time and aged 30–68 years (average age = 44.75 years). Most of the nursing experts were female (n = 34), and all participants possessed a Master of Science in Nursing degree. The demographic characteristics of the participants are shown in Table I.

All the participants completed each round of the Delphi method. The authors of this study analysed their responses and identified the elements of an effective PMEP. Subsequently, the authors created a list of elements that characterize an effective PMEP for nurses, which facilitates the implementation of such a programme. A successful Delphi study relies on the combined expertise of deliberately selected participants rather than the representativeness of sampling for statistical purposes. There are no universal rules governing the minimum and maximum numbers of participants. The heterogeneous panel of professionals selected for this study worked in hospitals, health centres, or as lecturers or instructors of nursing schools in the participants’ respective fields.

### Consensus

The authors of this study decided to consider questionnaires for all items with 75% or more agreement to measure the central trend of participants’ opinions. In the first round, 40 participants completed a questionnaire. The answers that were most relevant to enhancing nurses’ knowledge or their practice of pain management were identified. Subsequently, the authors analysed the responses, and following their discussion, ranked items based on their significance. In the second round, the authors sent the results of the first round to all participants to obtain their comments regarding the same. Subsequently, the authors reorganized the content based on the experts’ comments with new subheadings and determined what should be placed under each subheading.

A 5-point Likert scale was used to assess agreement between participants, ranging from strong agreement to strong disagreement. The questions were subsequently confirmed by three colleagues, who understood that the questions represented a high reporting rate. In the third round, participants answered questions that led to a list of items in each table based on their consensus and in the order of descending importance. The item with the highest percentage came first, and the lowest was placed at the very end; with the consensus power of respondents ranging from 87.5%—75%.

### Ethical consideration

The principles of ethical standards for conducting research using the Delphi technique were fully applied in this study (Hasson & Keeney, 2011; Kwon et al., 2020). General guidelines concerning informant consent and voluntary participation in recruiting informants to an expert panel were considered and applied in the study (Kwon et al., 2020). Although no formal ethical approval was required, principles for research ethics involving humans participating was the guiding principle throughout the process (sf. SFS, 2003; National Research Council of Thailand, 2015; World Medical Association, 2018). One of the authors provided the particulars of the research objective and methods of face-to-face research with the experts involved in the study. All experts provided formal consent upon receipt of the Delphi questionnaire. They were assured of anonymity and confidentiality of their information. It was made clear to them that they could withdraw from the study at any point while it was underway.

## Results

### The first round

Semi-structured interviews were used in the preparation of the first-round Delphi questionnaire with 40 experts who agreed on the number of participants (100%) in the study. The participants’ answers to the first-round questionnaire, including 402 items, were summarized and sorted by the content analysed in the second-round questionnaire (see Table II). Their answers from four categories were divided into three thematic sections: (i) elements that enhance the efficiency of a PMEP for nurses (67 responses), (ii) the main component of a PMEP for nurses (120 responses), and (iii) elements that could help nurses learn or improve their pain management practices (163 responses) and miscellaneous suggestions (52 responses).

**Table II.** Consensus generated in in the second round of the Delphi process.

Consensus of Questions	Item in each question
1) Name the elements that make a PMEP feasible and efficient for nurses. [11 out of 39 items]	1. Knowledge, attitudes, and beliefs of nurses regarding pain management practices.
	2. Ability to administer or teach consistent pain management programmes for nurses and to have ongoing knowledge of pain management.
	3. Nurses should be aware of the effects of PMEPs and positive attitudes towards good pain management in clinical practice.
	4. The nurses’ perception and response to the patient’s pain and a pain-relieving approach using nursing science.
	5. Nurses’ knowledge about pain, attitudes, and practices for assessing pain.
	6. The nurse must accept individual differences, including interpretation of pain signals and choice of appropriate method to manage pain.
	7. Managing pain with family support in terminally ill cancer patients.
	8. Evidence-based pain management models are an important part of nursing practice.
	9. The effect of an evidence-based PMEP on patient pain management outcomes and the development of clinical guidelines to manage pain in patients.
	10. The success of a PMEP requires multidisciplinary collaboration.
	11. Knowledge and innovation in clinical pain management are needed by the healthcare team.
2) In your opinion, what are the main components of a PMEP for nurses? [14 out of 23 items]	1. Assessment and recording of pain severity, type/nature of pain, pain management using different approaches/techniques/methods, and recording and evaluating pain management.
	2. Assessment of patient-related pain as this information can be used in care planning.
	3. Improving nurses’ knowledge and attitudes towards pain and organizational elements is a quality of care in managing pain.
	4. Knowledge of evidence-based techniques for pain management enhances nursing competence in the development of pain management models.
	5. Nurses are looking for innovations in pain management, including acute pain, chronic pain, and cancer-related terminal pain.
	6. Establishing clear objectives plans the systematic collaboration of the programme with a multidisciplinary team that tests the programme and reviews it if there are problems.
	7. The effectiveness of pain management and interdisciplinary collaboration is complemented by a training programme.
	8. The goal of pain management is to ensure the systematic monitoring of patients’ pain, with the goal of improving pain treatment.
	9. Guidelines on pain management and processing for reduction or relief of pain.
	10. Set up a protocol and programmes for managing pain in nurses. This programme should be short, concise, clear, easy to maintain and easy to use, leading to productivity improvements.
	11. Supervision and follow-up, feedback from PMEP users, and empowering nurses in PMEPs are needed. Such as patient satisfaction for current staff and identify who is using the PMEP.
	12. The education manual covers pain management, knowledge of evidence-based pain management, and assessing pain management.
	13. Demonstrating pain management procedures and the pain recording/monitoring form is the patient’s early admission.
	14. A pain management training programme should be accessible and easy to use.
3) Name at least three elements that could help nurses improve their competence in pain management. [7 out of 14 items]	1. Promoting the competency of professional nurses involves implementing an evidence-based pain management model and considering the implementation of the pain management programme.
	2. Structure the knowledge development system and skills according to the performance of a pain workshop to provide nurses with information on the cause and level of pain, in addition to finding ways to manage pain with accuracy and effectiveness.
	3. Appropriate mindset/attitude regarding pain management in nurses. Community nurses and nurse practitioners should provide insight into in-depth understanding of the factors and patients involved in the pain process.
	4. Nurse attitudes towards patients, knowledge of nursing educators and methods of knowledge transfer to nursing students, extending empathy to patients.
	5. Listening actively to patients when they are describing their pain and respecting their humanity.
	6. Recognizing the pain and appreciating the prospects for the patient and their family. It is important to care for the patients who are suffering. This might include strategies to educate nurses for providing pain relief throughout the labour period—touch massage strategies for pain relief during labour, and care and environmental management strategies for pain relief throughout the labour period.
	7. Family roles including patient care, the importance of pain management in patients with terminal cancer, and practices for the treatment of terminal pain.

**Figure 2.**
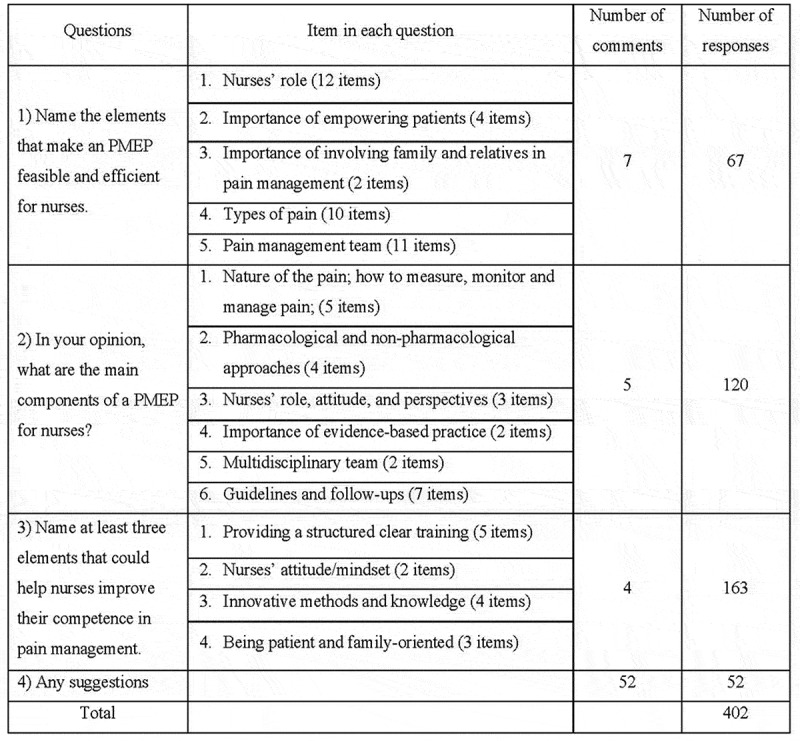
The first-round questionnaire.

Most experts who proposed the improvement of a PMEP also achieved consensus regarding the following three core themes: *(i) Elements that enhance the efficiency of a PMEP for nurses* (total 39 items, 7 comments were provided), *(ii) The main component of a PMEP for nurses* (total 23 items, five comments were provided), *(iii) Elements that could help nurses improve their competence in pain management* (a total of 14 items, four comments were provided), as shown in Figure 2.

### The second round

As described in the Methods section, the results from the first round were used to generate questions from the second round. The process consisted of 40 experts who completed the first-round questionnaires. The experts agreed on three core components to establish a PMEP for surgical nurses in Thailand. Each item in the questionnaire in the second round was rechecked and agreed/disagreed upon by the approved participants who agreed to more than 75% of the items. The experts agreed on the following core components: (i) elements that enhance the efficiency of a PMEP for nurses (11 out of 39 items), (ii) the main component of a PMEP for nurses (14 out of 23 items), and (iii) elements that could help nurses improve their competence in pain management (7 out of 14 items). The reasons for selecting these components were consistent for each part. According to expert responses, there is a need to recognize how nurses can improve their knowledge, attitudes, and beliefs regarding pain management skills or practice (see Table II).

### The third round

A consensus emerged among experts regarding the need for regular PMEP. Activities in such a PMEP provide nurses with information on how to treat problems related to patient pain. Most participants proposed a design for an effective PMEP for nurses. The experts also reached a consensus regarding the following five core clusters: (i) the success of the PMEP requires multidisciplinary collaboration; (ii) nurses’ knowledge, attitudes, and beliefs regarding the practice of assessing pain and pain management; (iii) healthcare teams need to possess knowledge and innovation regarding clinical pain management; (iv) nurses should be aware of the effects of PMEPs and develop positive attitudes towards efficient pain management in clinical practice; (v) nurses must accept patients’ individual differences, including the interpretation of pain signals, and choose an appropriate method to manage pain (see Table III).

**Table III.** Results of the third round: Elements enhancing the efficiency of a PMEP for nurses.

Statements	Percentage of agreement
1. The success of a PMEP requires multidisciplinary collaboration.	87.5%
2. Knowledge, attitudes, and beliefs of nurses regarding the practice of assessing pain and pain management.	85%
3. Healthcare teams need to possess knowledge and innovation regarding clinical pain management.	85%
4. Nurses should be aware of the effects of pain management programmes and develop positive attitudes towards efficient pain management in clinical practice.	82.5%
5. Nurses accept patients’ individual differences, including the interpretation of pain signals and choice of appropriate method to manage pain.	77.5%

A PMEP is an efficient method of implementing multidimensional activities. According to the suggestions of the nursing experts, a PMEP must have five core components: (i) A protocol and a programme to manage patients’ pain: This programme should be short, concise, clear, easy to maintain, and easy to use, leading to productivity improvements. (ii) Supervision, follow-up, feedback, and evaluation: Patients must be asked for feedback regarding their satisfaction with the current staff, and the effectiveness of the PMEP in improving nurses’ competence should be assessed. (iii) A complementary training programme: Training must be provided to enhance the effectiveness of pain management practices and interdisciplinary collaboration. (iv) Demonstrations and early monitoring: Demonstrations of pain management procedures must be provided to nurses, and the pain experienced by patients must be monitored from the moment of admission into ward. (v) Accessibility and ease of use (see Table IV).

**Table IV.** Results of the third round—Main components of a PMEP for nurses.

Statements	Percentage of agreement
1. Setting up a protocol and programmes for nurses to manage patients’ pain. This programme should be short, concise, clear, easy to maintain, and easy to use, leading to productivity improvements.	82.5%
2. Supervision and follow-up, feedback from pain management programme users, and empowering nurses regarding pain management programmes are needed. This includes assessing patient satisfaction regarding the current staff and identifying who is using the PMEP.	82.5%
3. The effectiveness of pain management and interdisciplinary collaboration should be complemented by a training programme.	80%
4. Pain management procedures should be demonstrated to nurses and the pain recording/monitoring of a patient since their early admission should be conducted.	80%
5. A pain management training programme should be accessible and easy to use.	80%

Additionally, most participants proposed elements that would help nurses learn about new pain management practices or improve the ones already in use. The experts reached a consensus on how the PMEP could tackle the core training needs of nurses and equip them with the skills essential and suitable for pain management. They agreed that the programme should target the following five major areas: (i) family roles, including patient care, the importance of pain management in patients with terminal cancer, and practices for the treatment of terminal pain; (ii) actively listening to patients when they are describing their pain and respecting their humanity; (iii) promoting the competency of professional nurses by implementing an evidence-based pain management model and considering the implementation of a PMEP; (iv) nurses’ attitudes towards patients, knowledge of nursing educators and methods of this transfer of knowledge to nursing students, and extending empathy to patients; and (v) structuring the knowledge development system and skills according to the performance of a pain workshop to provide nurses with information on the cause and level of pain, in addition to assisting them in developing ways to manage pain accurately and effectively (Table V).

**Table V.** Results of the third round—Elements with the potential to help nurses to learn/improve their pain management practices.

Statements	Percentage of agreement
1. Family roles include patient care, the importance of pain management in patients with terminal cancer, and practices for the treatment of terminal pain.	85%
2. Actively listening to patients when they are describing their pain and respecting their humanity.	82.5%
3. Promoting the competency of professional nurses involves implementing an evidence-based pain management model and considering the implementation of a PMEP.	82.5%
4. Nurse attitudes towards patients, knowledge of nursing educators and methods of transfer of this knowledge to nursing students and extending empathy to patients.	80%
5. Structure the knowledge development system and skills according to the performance of a pain workshop to provide nurses with information on the cause and level of pain, in addition to assisting them in developing ways to manage pain with accuracy and effectiveness.	77.5%

## Discussion

This study found consensus among experts regarding the core components necessary for developing a PMEP for nurses. Previous studies found that postoperative pain management is poor in Thailand, which is mainly attributed to inefficient PMEPs (Jaleta et al., 2021; Al Samaraee et al., 2010). The experts in this study proposed some components to enhance PMEP effectiveness. According to them, an effective PMEP should include the following three core components: (i) multidisciplinary collaboration, (ii) innovative knowledge and training on pain management for healthcare teams, and (iii) consideration of individual differences when delivering pain management services. The experts agreed that the value provided by these components matched the quality standards and recognition processes established by professionals. Nurses who have completed approved programmes and have undergone rigorous training and education can help inspire confidence in health policy and hospital leadership (Coll & Jones, 2020; Hu et al., 2017; Jaleta et al., 2021; Al Samaraee et al., 2010; Tano et al., 2021).

Our study used a consensus-based approach to determine the core components of a PMEP for nurses, leading to the formation of a framework for effective PMEP. The experts mentioned a protocol and elements of PMEPs related to nurses’ knowledge and attitudes regarding pain assessment and effective pain management. The standard for PMEPs is to make these programmes accessible and easily usable, resulting in the maintenance of the delivery of patient pain management services. The experts in this study sought to improve the effectiveness of pain management and interdisciplinary collaboration (Coll & Jones, 2020; Keen et al., 2017; Al Samaraee et al., 2010). One component was the inclusion of pain education in nursing education, supported by evidence such as psychoeducation, strategies for enhanced activity, and cognitive approaches (Kwon et al., 2020). However, these findings are similar to those of other studies conducted by professional nurses with appropriate training in pain management. Nurses’ attitudes towards patients, knowledge of nursing educators, methods of transferring this knowledge to nursing students, and extending empathy to patients can be substantiated. However, nurses’ PMEPs are wide-ranging, and the duration of the programme, faculty requirements, and higher qualifications differ in different countries. Healthcare services can be effectively implemented with proper PMEPs supported by robust policies, guidelines, and up-to-date evidence (Keen et al., 2017; Coll & Jones, 2020; Hu et al., 2021; Hyland et al., 2021; Ramamoorthy, 2021; Tano et al., 2021)

The Delphi method has been extensively applied in nursing education research, including defining competencies, developing curricula, and developing methods or tools to evaluate learning programmes (Basinska et al., 2021; Hu et al., 2017; Sasahara et al., 2009; Sharpe et al., 2020). Additionally, this study is relevant to the development of PMEPs. Nurses play a role in the development of a nursing curriculum model by identifying and linking the scope of practice, skills, curricula, programme requirements, and internationally recognized training standards. The Delphi technique used in this study is based on the standard guidelines outlined by Keeney et al. (2011). These guidelines were employed in the present study, including preparation of the initial questionnaire, selection of participants, and consensus definitions. Hence, the validity of the methods and results of this study were ensured. This study can inform the best practices of nurses studying in hospitals, health centres, or teachers/instructors in nursing schools.

## Conclusion

This research provides a foundation for building an effective PMEP for healthcare nurses. The experts in this study agreed on several subjects that include three 5-item categories: (i) elements that enhance the efficiency of a PMEP, (ii) the main component of a PMEP for nurses, and (iii) elements that could help nurses improve their pain management practices. These elements were identified for improving PMEPs. The resulting training programme containing these elements will reflect nursing education standards.

The results of this study will be useful in developing countermeasures for pain as part of a PMEP for professional nurses. The insight and knowledge provided by the study can equip nurses; in particular surgical nurses in managing the post-operative pain in an effective way. The surgical nurses in Thailand need such support to shift from the current passive approach to pain management to a comprehensive approach, suggested by this study.

## Authors’ contributions

The first author collected and analysed the data, interpreted the findings, and wrote the manuscript. The other authors helped design the study and collect data. They wrote and revised the sections of the manuscript. The final manuscript was reviewed and approved by all authors.
